# Rescue Revascularisation in Acute Internal Carotid Artery Occlusion with a Super Extended Time Window of More than 48 hours

**DOI:** 10.1155/2022/9036082

**Published:** 2022-04-30

**Authors:** Katharina Berger, Jennifer Sartor-Pfeiffer, Annerose Mengel, Ulrike Ernemann, Ulf Ziemann, Florian Hennersdorf, Katharina Feil

**Affiliations:** ^1^Centre for Neurovascular Diseases Tübingen, ZNET: University of Tübingen, Tübingen, Germany; ^2^Department of Neurology & Epileptology, University of Tübingen, Tübingen, Germany; ^3^Hertie Institute for Clinical Brain Research, University Tübingen, Tübingen, Germany; ^4^Department of Neurology & Stroke, University of Tübingen, Tübingen, Germany; ^5^Department of Diagnostic & Interventional Neuroradiology, University of Tübingen, Tübingen, Germany

## Abstract

**Methods:**

We present the case of a 71-year-old Caucasian male “minor stroke” patient with LVO, good collateral flow via the ophthalmic artery, receiving rescue MT following clinical deterioration after >48 hours. NIHSS and modified Rankin scale (mRS) were used for follow-up and modified treatment in cerebral infarction (mTICI) score for angiographic results.

**Results:**

Excellent angiographic result (mTICI 3) and clinical improvement were achieved (NIHSS preintervention 18, on discharge 2 points). 90-day follow-up showed excellent outcome (mRS 1).

**Conclusions:**

Late intervention MT should be encouraged when clinical deficit exceeds infarct demarcation. Standardized identification based on clinical and imaging data is required to target critical patients with LVO and low NIHSS, favouring a primary intervention.

## 1. Introduction

Mechanical thrombectomy (MT) in combination with intravenous thrombolysis (IVT) has become the gold standard treatment for stroke patients caused by large vessel occlusion (LVO) [1]. Although LVO typically leads to severe stroke, at least 10–20% of stroke patients presenting with LVO have only mild symptoms (National Institutes of Health Stroke Scale (NIHSS) ≤5, i.e., minor stroke). These patients are at high risk of clinical neurological deterioration and poor outcome [1, 2]. Therefore, treatment guidelines in minor stroke patients remain controversial [3]. Whilst recanalization rates in LVO with recombinant tissue plasminogen activator (rtPA) alone are unsatisfactory, endovascular treatment can be associated with higher mortality rates due to the increased risk of symptomatic intracerebral hemorrhage [4]. Recent observational studies comparing immediate MT in these patients and best medical therapy followed by rescue MT in deteriorating cases have shown superior outcomes following immediate MT in minor stroke patients [2]. Current guidelines suggest MT within 6 hours, with the option for late window interventions after 6 hours [1]. Results given by the DEFUSE3 and DAWN studies also encourage late interventions in selected patients [1, 5]. The decision for or against MT in LVO patients with minor stroke symptoms and clinical deterioration is currently based on interdisciplinary single case discussion. Predictive measures which determine the patient's risk for deterioration in case of withholding MT are not established. Here, we report on a case of a super extended time window of more than 48 hours after symptom onset until rescue MT was performed.

## 2. Case Report

**Figure 1:**
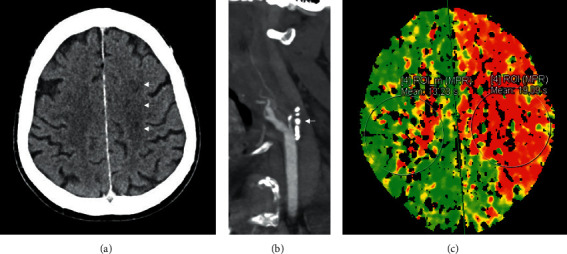
(a) Noncontrast CT: small border zone infarcts (white arrows) ASPECTS 10. (b) CT angiography: occlusion of the left ICA caused by a preexisting atherosclerotic stenosis (white arrow). (c) Time-to-peak map of CT perfusion: delay in maximum attenuation of 5.7 seconds.

A 71-year-old Caucasian male patient (premorbid modified Rankin scale (pmRS) 0), with a medical history of hypertension, presented with aphasia and right hemiparesis (National Institutes of Health Stroke Scale (NIHSS) 8 points) in a regional hospital; symptoms had persisted for more than six hours at time of admission. Initial cerebral computed tomography (cCT) with CT angiography (CTA) showed no infarct demarcation, but proximal LVO of the left internal carotid artery (ICA). Due to the expired time window, IVT was not performed, and the patient was transferred via “drip and ship” to the Center of Neurovascular Diseases of the University Hospital of Tuebingen (ZNET). On in-house admission, the patient presented with only a mild neurological deficit (NIHSS 3 points given for Broca's aphasia and mild right arm paresis). cCT with CTA and CT perfusion (CTP) showed a persistent occlusion of the left ICA, with beginning border zone infarct demarcation between the anterior and middle cerebral artery territories and an extensive perfusion deficit within the territory of the middle and anterior cerebral arteries with a delay in a time-to-peak of 5.7 s in comparison to the nonaffected hemisphere (Figure 1).

Due to the low NIHSS on admission, the late time window, as well as the assumed collateralization (due to only minor neurological symptoms), a decision was made in favour of a conservative treatment, and invasive blood pressure measurement was initiated for monitoring. The patient initially remained clinically stable for more than 24 hours after stroke onset. The patient received a dual antiplatelet and high-dose statin-therapy with atorvastatin. Afterwards, he began to develop fluctuating neurological symptoms dependent on variations in systolic blood pressure. In follow-up imaging, the ICA remained occluded, with a reduced blood flow in the middle cerebral artery (MCA) and a retrograde blood flow in the ophthalmic artery in duplex sonography.

Any systolic blood pressure dropping below 230 mmHg resulted in global aphasia, as well as right-sided hemiplegia (NIHSS 18 points), whereas systolic blood pressure levels above 230 mmHg resulted in immediate symptom improvement (minimal NIHSS 3 points), indicating that collateral blood flow was maintained only through high perfusion pressure. Further investigations showed that the cerebral infarct demarcation remained the same as on initial presentation with a persisting perfusion deficit indicating a small core infarct with a large volume of tissue at risk. Despite the extended time window of more than 48 hours after stroke symptom onset, an interdisciplinary decision for off-label rescue MT was made. No drugs with a potential blood pressure lowering effect were administered prior to intervention.

**Figure 2:**
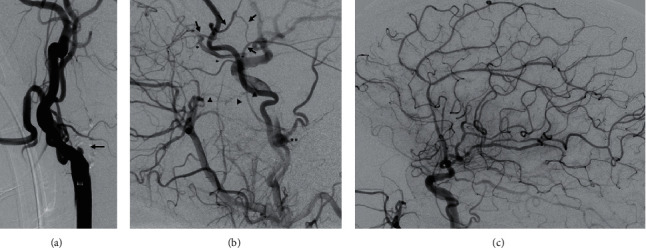
(a) Lateral angiogram of the common carotid artery (CCA) showing occlusion of the ICA. (b) Lateral CCA angiogram: exclusive filling of the intracranial vessels through multiple collaterals (^*∗*^), retrogradely filled ophthalmic artery (OA); arrows depict a connection between the middle meningeal artery (MMA) and OA. Dural anastomoses from MMA to ICA at the level of the skull base (^*∗∗*^); arrow heads depict a branch of the sphenopalatine artery feeding the cavernous ICA (superimposed by the superficial temporal artery). (c) Lateral angiogram after intervention: (TICI3). Vessel wall irregularities are rated as atherosclerotic.

Angiograms of the left common carotid artery showed the occluded ICA with distal filling at the level of the skull base through multiple collaterals from the external carotid artery indicating insufficient intracranial crossflow (Figures 2(a) and 2(b)). The procedure has been performed under general anaesthesia, using an 8F balloon guide catheter (Corail Plus™, Balt). The occluded vessel was passed with a microwire, and angioplasty was performed using a percutaneous transluminal angioplasty (PTA) catheter. Thromboaspiration was performed on site of the protruded guider, enabling revascularisation of the left ICA, however, with a remaining high-grade stenosis of the revascularized vessel. The remaining stenosis was then treated by placement of a stent (Precise Pro RX™, Cardinal Health).

Despite concerns about thromboembolism from the long-term persisting long-distance occlusion, the final angiograms showed no intracranial vessel occlusions (modified treatment in cerebral infarction (mTICI) score 3) (Figure 2(c)). The post-MT cCT did not show any further infarct demarcation or secondary intracranial hemorrhage.

Thereafter, the patient's clinical presentation improved significantly; blood pressure values normalized into the high-normal range. The patient was discharged to neurological rehabilitation with NIHSS 2 points (mRS 2) given for disorientation in time and space, due to a hyperactive delirium, which occurred as a complication. In the further clinical course at 90-day follow-up, the patient had an excellent clinical outcome (mRS 1).

## 3. Discussion

Our case presents a patient with minor stroke despite proximal LVO of the ICA turning to massive clinical deterioration after initial decision for conservative treatment. Although efficacy of MT is time-dependent [1, 2], the results of DAWN and DIFFUSE3 showed that selected patients benefit from MT in an extended time window of 6–24 hours. In our case, this window has been exceeded by far, based on a second cCT presenting still without clear infarct demarcation except for small watershed infarctions (Alberta stroke program early CT score (ASPECTS) 10) and extensive tissue at risk with only small core infarct volume on CTP. Despite concerns about thromboembolism from the long-term persisting, long-distance occlusion, the procedure was successfully performed with an excellent angiographic result (mTICI 3) and clinical outcome, and postinterventional imaging showed no increase in infarct volume.

Studies suggest that a preexisting good collateral flow is predictive for a favourable outcome after MT and can be also used as a selection criterion for patients [6]. In the present case, the clinical deficits fluctuated in association with blood pressure variation, indicating that the cerebral perfusion provided by the collateral blood flow (Figure 2(b)) was insufficient and maintained only through high perfusion pressure as any systolic blood pressure dropping below 230 mmHg resulted in a massive clinical deterioration with NIHSS increasing from 3 to 18 points. Taking the results of DEFUSE3 and DAWN into consideration, collateral blood flow has not been a selection criterion for or against MT. Contrary, criteria for intervention were LVO in the anterior circulation, small infarct volume, as well as high NIHSS. In summary, despite the late time window, our patient fulfilled the described criteria favouring MT.

Furthermore, this case once more highlights that the NIHSS cannot alone be used as a score to evaluate which primary treatment is the most appropriate regarding LVO, although to date, it is the only validated clinical score used for decision making, especially regarding rescue MT [7]. Currently, there are no clear clinical indicators, in which minor stroke patients are at high risk of clinical deterioration and accordingly would likely benefit from primary intervention. Lately, possible scores based on thrombus length and site of occlusion were discussed as easily applicable but require clinical validation [6].

## 4. Conclusion

Late window MT should be considered in patients with minor stroke and LVO based on standardized criteria. Especially in the context of clinical deterioration and the clinical deficit exceeding the infarct demarcation but rather matching the perfusion deficit, a reevaluation towards MT should be made. A high initial NIHSS score in the context of early improvement can imply a high likelihood of secondary deterioration as it stresses the significance of the occlusion and the clinical impact in case of collateral circulation break down. A standardized procedure based on clinical and imaging data might help to identify such critical patients for early reperfusion. Prospective studies are required for confirmation and validation.

## Data Availability

The data generated or analysed during this study are included within the article and are available from the corresponding author upon request.
